# Doping Prevalence in Sport from Indirect Estimation Models: A Systematic Review and Meta-analysis

**DOI:** 10.1186/s40798-026-01014-z

**Published:** 2026-06-07

**Authors:** Dominic Sagoe, Maarten Cruyff, Razieh Chegeni, Annalena Veltmaat, Anna Kiss, Sándor Soós, Olivier de Hon, Peter van der Heijden, Andrea Petróczi

**Affiliations:** 1 Department of Psychosocial Science, University of Bergen, Christiesgate 12, 5015 Bergen, Norway; 2 Human Enhancement and Body Image Lab (HEBI Lab), Addiction Research Group, University of Bergen, Bergen, Norway; 3 Faculty of Social Sciences, Utrecht University, Utrecht, Netherlands; 4 Department of Psychology, PROMENTA Research Center, University of Oslo, Oslo, Norway; 5 Department of Sport and Sports Science, TU Dortmund University, Dortmund, Germany; 6 Department of Science Policy and Scientometrics, Hungarian Academy of Sciences (MTA), Budapest, Hungary; 7 Faculty of Education and Psychology, Eötvös Loránd University (ELTE), Budapest, Hungary; 8 Doping Authority Netherlands, Capelle aan den IJssel, Netherlands; 9 Southampton Statistical Sciences Research Institute, University of Southampton, Southampton, UK; 10 Faculty of Health and Sport Sciences, Széchenyi István University, Győr, Hungary

**Keywords:** Doping, Indirect estimation models, Prevalence, Randomised response techniques, Sport

## Abstract

**Background:**

To our knowledge, no previous systematic review and meta-analysis of doping prevalence in sport from indirect estimation models (IEM) exists.

**Objective:**

To conduct a systematic review and meta-analysis of empirical IEM-based studies of admitted doping prevalence in sport.

**Methods:**

We conducted electronic database and ad hoc searches up to March 2025, and estimated lifetime and past year prevalence rates through a cross-classified model including prevalence (lifetime vs. past year), sample (competitive vs. recreational) and sports (multi-sport vs. single-sport) types.

**Results:**

Forty-six records (*K*) were included in the review (*k* [subset records included in the meta-analysis] = 30, *n* [independent studies from the records] = 34). The World Anti-Doping Agency’s definition of doping use was applied for data collection in most studies (*k* = 18), and doping prevalence was mostly assessed as past year/season (*k* = 20). Studies included in the meta-analysis were mostly conducted in Europe (*k* = 22) and applied the Unrelated Question (*k* = 8) and Forced Response with Cheater Detection (*k* = 6) models. Study participants were mostly multi-sport (*k* = 20) and competed at diverse levels, and most data (*k* = 28) was collected outside sport events. The corpus included articles that re-analysed existing data (*k* = 4). Lifetime prevalence was highest for multi-sport competitive athletes (22.6%) and lowest for single-sport competitive athletes (12.7%), whereas past year prevalence was highest for single-sport recreational sportspersons (15.5%) and lowest for multi-sport recreational sportspersons (8.7%).

**Conclusions:**

Under IEM, about one of five multi-sport competitive athletes admitted to ever doping whereas about one of six of single-sport recreational sportspersons admitted to doping in the past year. Furthermore, multi-sport (vs. single-sport) competitive athletes show relatively higher doping prevalences, whereas single-sport (vs. multi-sport) recreational sportspersons report relatively higher doping prevalences. Secondary (re-)analysis presents a novel methodological challenge for meta-analyses.

*Registration* PROSPERO: CRD42022373691.

**Supplementary Information:**

The online version contains supplementary material available at 10.1186/s40798-026-01014-z.

## Key Points

- Under indirect estimation models, about one of five multi-sport competitive athletes admits to ever doping.
- About one of six of single-sport recreational sportspersons admits to doping in the past year.
- Multi-sport (vs. single-sport) competitive athletes show relatively higher doping prevalences.
- Single-sport (vs. multi-sport) recreational sportspersons report relatively higher doping prevalences.
- Secondary data analysis, often to improve analytical procedures, presents a novel challenge for meta-analyses.

**Supplementary Information:**

The online version contains supplementary material available at 10.1186/s40798-026-01014-z.

## Introduction

Estimating the prevalence of doping, operationalised as the use of prohibited substances and/or methods without therapeutic use exemption, is vital for sport for multiple reasons. First, doping prevalence reflects the burden of doping in a given population, such as a sport, a country or a major event. This burden is not limited to the costs of doping control but includes the detrimental impact on the sport, the athletes, event organisers, supporters, sponsors, broadcasters, sport equipment and apparel manufacturers. Second, knowing the prevalence of doping assists policy-makers in determining where investments in detection and prevention should be targeted. Such knowledge is also useful for formulating preventive measures and methods for early detection, as well as for monitoring programmes for evaluating the effectiveness of the measures put in place to reduce prevalence and incidence rates.

To estimate doping prevalence in sport, researchers and practitioners have relied on self-reported surveys which, although cost-effective and feasible for large representative samples, have specific limitations, such as dishonest and socially desirable responding [1–3]. To address this limitation, anti-doping researchers and organisations have turned to Indirect Estimation Models (IEM), such as the Randomised Response Technique (RRT) [3] surveys, as they offer protection over and above anonymity due to their unique design [4]. Examples of RRT are the Forced Response (FR) [5], Kuk’s Design [6], the Unrelated Question Model (UQM) [7, 8], the Crosswise Model (CM) [9, 10, 13] and the Single Sample Count (SSC) [84]. However, to our knowledge, no previous systematic review and meta-analysis studies using IEM—which allows for pooling smaller samples segmented by countries, sports and different levels of competition together for estimating the global prevalence of doping in sport—has been conducted. Therefore, in the present study we first systematically reviewed research outputs using one or more IEM to estimate the prevalence of doping in sport, and then quantitatively synthesised these findings using meta-analysis.

## Methods

### Registration, Search Strategy and Inclusion Criteria

The study was pre-registered in PROSPERO (CRD42022373691).

 A systematic literature search in the ProQuest, PsycNET, PubMed, Web of Science and Google Scholar databases was performed using the following keywords: “doping OR anabolic OR prohibited AND prevalence OR estimat* AND model OR response”. The same search was conducted in German in the SPORTDiscus, SPONET, BISp-Surf, Scopus, Web of Science and Google Scholar databases. The English literature search was conducted by DS and the German literature search by AV. Additionally, automated searches were conducted by AK and SS in French, Russian and Spanish.

 The key inclusion criteria were studies: (1) using indirect (randomised and non-random) estimation models in determining doping prevalence in sport, and (2) publication in English, Dutch, German, French, Russian or Spanish. Ad hoc searches were also conducted in the OpenGrey (SIGLE) database and reference lists for grey literature and for comprehensiveness assurance. The latest database literature search was conducted in March 2025. The literature search and selection was conducted using the Preferred Reporting Items for Systematic Reviews and Meta-Analyses (PRISMA) procedure [11].

### Data Extraction and Synthesis

Using a standardised data extraction form, the following data were extracted from the identified studies: author(s) and publication year, model used, sample (size, country, sport, competition level, age range, expressed as the mean ± standard deviation [M ± SD]) and data (collection method, year, settings and if the World Anti-Doping Agency [WADA] code definition of doping was applied) characteristics, estimated doping prevalence, non-compliance assessment, doping definition, prevalence timeframe, validation and response rate (Additional file 1: Table S1). Using content analysis [12], the first author (DS) extracted the data and selected the relevant articles based on the inclusion criteria, with the last author (AP) providing data from a WADA (2022 – unpublished data)^1^ project.

### Meta-analysis

A meta-analysis was conducted to estimate doping prevalence through a cross-classification of prevalence type, distinguishing between lifetime (“ever”) and past year use (“current season, past 3 months, last year, past season and past year”); sample type, categorised as competitive athletes (“competitive, elite, international, national, regional, schoolboy, university student athletes”) and recreational sportspersons (“gym goers, recreational”); and sports type (multi-sport vs. single-sport events).

Initially, when we encountered studies with duplicate data, such as studies in German [24, 25] and a study in English [26], we relied on data extracted from one published study [26] to avoid duplicate data extraction and analyses. We excluded studies comprising a mix of competitive and recreational samples. Also, we separated lifetime and past year prevalences in line with the empirical data although, by definition, lifetime use of doping comprises current and past year use. The analysis includes 55 prevalence estimates nested within 34 empirical IEM studies. Due to the hierarchical structure of the data, we adopted the procedure of Lensvelt-Mulders et al. [14], and conducted a weighted multilevel analysis with the 55 doping prevalence estimates at level 1 and the 34 empirical IEM studies at level 2. To normalise the dependent variable we converted the prevalence estimates from the probability scale to the probit scale by computing their z-scores. To account for the heterogeneity of the prevalence estimates, we included prevalence type (lifetime vs. past year), sample type (competitive vs. recreational) and sports type (single-sport vs. multi-sport) in the multilevel model as predictors, with interaction terms for sports type and prevalence type as well as sample type.

To account for the heterogeneity in the precision of the prevalence estimates, we weighted each estimate by the inverse of its estimated variance, so that estimates with narrow confidence intervals are weighted more heavily in the analysis than estimates with a wide confidence interval. A special case in this respect are the prevalence estimates obtained with the Cheater Detection Model (CDM) [15], which yields a lower bound estimate of “honest” doping users and an upper bound of “honest” doping users and “cheaters”, whose status with respect to doping use is unknown. In order to use the results of these studies in the meta-analysis, we used the midpoint of the lower and upper bound as the point estimate of doping use and then computed the variance as the compound variance of the lower and upper bound plus the variance of the point estimates of “honest” doping users and “cheaters”. By taking the midpoint we assume that half of the cheaters use doping, and the other half do not. This will lead to an underestimate of the doping prevalence if the majority of cheaters use doping, and to an underestimate if the opposite is the case. The meta-analysis was conducted using R version 4.0.5 [16] with the lme4 [17] and tidyverse [18] packages.

## Results

### Study Selection

**Fig. 1:**
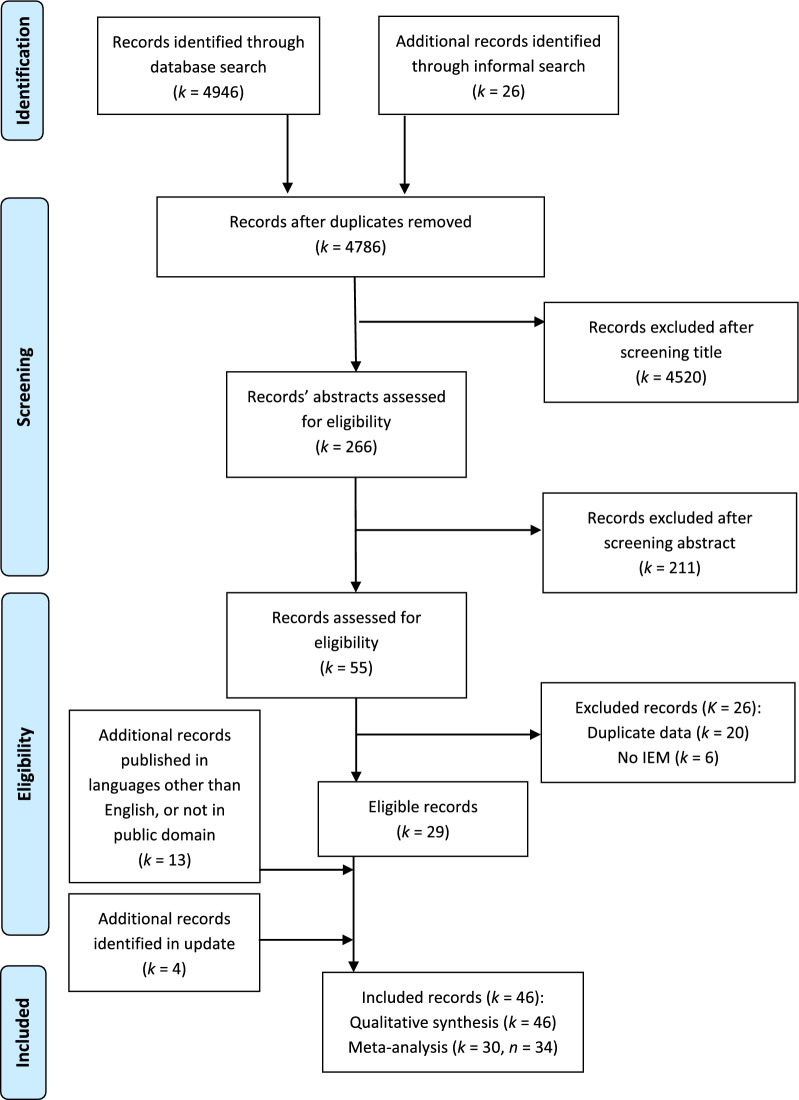
Flow diagram of systematic literature search. IEM Indirect Estimation Model

A total of 4,946 hits were identified from the database search and 25 records through an informal search comprising the authors’ sources and reference list checks. Of these, 4,786 records remained after removing duplicates, with 4,520 records deleted after title screening. Through abstract screening of the remaining 266 records, 211 records were excluded. Of the remaining 55 records assessed for eligibility, 26 records were excluded due to duplicate data (*k* = 20) and absence of IEM (*k* = 6). Thus, 29 full-text records met our eligibility criteria. Also, 13 additional records were identified through searches in other languages and by consulting experts in the field, and four additional records were identified during an updated search during the peer-review process. This yielded a total of 46 records included in the review. The results of the literature search and the steps in the selection process are shown in Fig. 1.

### Overview of Outputs

Due to the heterogeneity of the included papers, albeit consistent with previous doping meta-analyses [19–23], not all outputs were included in all analyses. These are detailed in Table 1. All records (*K* = 46) were included in the qualitative synthesis, and a subset (*k* [records] = 30, *n* [independent studies from the records] = 34) were included in the meta-analysis.

**Table 1:** Data structure for the different analyses presented in the systematic review

Study^a^	Language	First author’s country of affiliation	Sample	Overview (qualitative synthesis)^b^	Meta-analysis^b^
Abdulrazzaq and Tareq [71]	English	Iraq	Recreational	○	○
Anti-Doping Agency of Serbia [47]	English	Serbia	Competitive	○	○
Backhouse et al. [72]	English	UK	Competitive	○	○
Balk and Dopheide [31]a	Dutch	Netherlands	Competitive	○	□
Balk et al. [32]a	English	Netherlands	Competitive	○	○
Boardley et al. [39]	English	UK^f^	Competitive	○	○
Breuer and Hallmann [48]	German	Germany	Competitive	○	○
Christiansen et al. [37]b	English	Denmark	Mixed, mostly recreational	○	□
Cruyff et al. [38]	English	Netherlands^f^	Competitive	○	○
Dietz et al. [40]	English	Germany	Recreational	○	○
Dietz et al. [51]	English	Germany	Competitive	○	□
Duiven and de Hon [30]^c^	Dutch	Netherlands	Competitive	○	○
Elbe and Pitsch [42]	English	Germany	Competitive	○	○
Fincoeur and Pitsch [49]	Dutch	Belgium†	Competitive	○	○
Franke et al. [73]	English	Germany	Competitive	○	○
Frenger et al. [74]	English	Germany	Mixed, mostly recreational	○	□
Heller et al. [75]	English	Germany	Recreational	○	□
Heyes [43]	English	UK	Competitive	○	○
Hilkens et al. [76]	English	Netherlands	Recreational	○	○
James et al. [41]	English	UK	Competitive	○	○
Musch and Plessner (2002 – personal communication)	English	Germany	Competitive	○	○
Nakhaee et al. [77]	English	Iran	Mixed	○	□
Nilaweera et al. [78]	English	Sri Lanka	Competitive	○	○
Petróczi et al. [34]^d,e^	English	UK^f^	Competitive	○	○
Pitsch [79]	English	Germany	Mixed	○	□
Pitsch [36]b	English	Germany	Mixed, mostly recreational	○	○
Pitsch and Emrich [26]d	English	Germany	Competitive	○	○
Pitsch et al. [27]b	German	Germany	Competitive	○	□
Pitsch et al. [28]b	English	Germany	Competitive	○	○
Pitsch et al. [24]d	German	Germany	Competitive	○	□
Plessner and Musch [29]c	German	Germany	Competitive	○	□
Reiber et al. [86]	English	Germany	Competitive	○	□
Robach et al. [89]	English	France	Competitive	○	○
Sayed et al. [44]	English	Netherlands	Mixed, mostly recreational	○	○
Sayed et al. [90]	English	Netherlands	Mixed	○	□
Sayed et al. [90]	English	Netherlands	Mixed	○	□
Schröter et al. [46]	English	Germany	Recreational	○	○
Seifarth et al. [45]	English	Germany	Recreational	○	○
Simon et al. [54]	English	Germany	Recreational	○	○
Stamm et al. [81]	German	Germany	Competitive	○	□
Striegel [82]	German	Germany	Recreational	○	□
Striegel et al. [50]	English	Germany	Recreational	○	○
Stubbe et al. [83]	English	Germany	Recreational	○	○
Ulrich et al. [33]^d^	English	Germany	Competitive	○	○
Ulrich et al. [35]^e^^,f^	English	Germany	Competitive	○	□
WADA (2022 – unpublished data)	English	UK^f^	Competitive	○	○

^a^Studies followed by the same lowercase letter (a, b, c, d) used the same data

^b^Open circle (○): included; open square (□): not included

^c^Published in English, summary available

^d^Studies used the same population

^e^Re-analysis of same data

^f^International collaboration

Of the 46 records identified, most were published in English (*k* = 37), followed by German (*k* = 6), and Dutch (*k* = 3). A notable overlap and duplication were found between the English and German language versions. Specifically, two studies in German [24, 25] present the same studies and data as in Pitsch and Emrich [26] in English, and also contain data from an earlier study [27] which was also reported in another study [28]. In the latter studies, Pitsch et al. [24, 25] explained that they conducted the replication study in response to the criticism about the chosen method in their first study design. Data from an unpublished manuscript (Musch and Plessner (2002 – personal communication))^2^, included with permission, were presented in a conference abstract [29] in English. Additionally, results from the more recent doping prevalence study in the Netherlands [30, 31] were published in Balk et al. [32]. Studies by Ulrich et al. [33] and Petróczi et al. [34] were conducted in the same settings (and with the same sample for one set of data) but using different IEM, whereas Ulrich et al. [35] present a re-analysis of the data reported in Ulrich et al. [33] and Petróczi et al. [34]. Lastly, Pitsch [36] and Christiansen et al. [37] report the same study.

Outputs were dominantly research articles (*k* = 30), followed by book chapters (*k* = 5), publicly available reports (*k* = 4), unpublished research report (*k* = 2), published conference abstracts (*k* = 2), magazine article (*k* = 1), PhD thesis (*k* = 1) and an unpublished manuscript (*k* = 1). Over 60% of the outputs focused on competitive sport at levels ranging from local club to elite international but the duplicate publications inflate this number (see Additional file 1: Table S1).

### Study Characteristics

#### Publication Years and Origin

Of the studies included in the qualitative synthesis and meta-analysis, publication years ranged from 2002 (Musch and Plessner (2002 – personal communication)) to 2024 [38, 88–90]. Similar to the full set of outputs, the meta-analysis studies were mostly conducted in Germany (*k* = 10), followed by the Netherlands (*k* = 4) and the UK (*k* = 3), with one study each from Belgium, Denmark, France, Iran, Iraq, Serbia, Sri Lanka, the USA and unspecified countries in a multi-study paper [38]. There were eight international studies [33, 34, 36, 38–41] (see Additional file 1: Table S1).

#### Participants

A total of 46,052 participants (specified *n*: males = 16,046, females = 3,118) participated in the 34 studies included in the meta-analysis. Sample sizes ranged from 249 [32] to 4,629 (WADA (2022 – unpublished data)) with a mean of 1,354.47 (SD = 1107.43) and were justified by power analysis in seven studies [38, 40, 42–45, 89].

Study participants were preponderantly multi-sport (*n* = 22). Studies also sampled triathlon (*n* = 3), bodybuilding (*n* = 2), cycling (*n* = 2) and track and field (*n* = 2) athletes as well as gym goers (*n* = 2) and chess players (*n* = 1). Participants’ competition levels comprised national (*n* = 11), international (*n* = 6), diverse (e.g. international, regional, national, local and recreational, *n* = 6), local (*n* = 3), recreational (*n* = 3), regional (*n* = 1), university (*n* = 1), schoolboy (*n* = 1) and “competitive” (*n* = 2) (see Additional file 1: Table S1).

#### Estimation Models

Most studies included in the meta-analysis applied the UQM (*k* = 8) followed by the FR with CDM (*k* = 6) and without the CDM (*k* = 3). Others were the CM (*k* = 4), Kuk’s Design (*k* = 3), SSC (*k* = 2), UQM and FR with CDM (*k* = 2) and UQM and SSC (*k* = 1). Nine outputs each [26, 28, 36, 41, 42, 46, 74, 79] presented results from multiple models. A comparison of these IEMS is presented in Table 2.

**Table 2:** Comparison of the indirect estimation models used in doping prevalence studies

Model comparison	Crosswise model (CM) [9]	Forced response (FR) technique [5]	Kuk’s Design [6]	Single sample count (SSC) [84]	Unrelated question model (UQM) [7]
Short description	The model combines two questions (one sensitive target question and one innocuous question with known probability). Respondents are instructed to answer the two questions together by only revealing if they have two of the same answers (yes–yes or no–no) or two different answers (yes–no or no–yes) without specifying which one exactly	The model uses a randomiser to instruct participants on which one of the three options to follow: one option is to answer the sensitive target question, one option forces respondent to say ‘yes’/’true’, and one option forces respondents to say ‘no’/’false’. In some variants, forced ‘yes’ and forced ‘no’ is replaced by statements that are obviously ‘true’ or ‘false’	In Warner’s model [3], the respondent answers one of two complementary statements, e.g. “I have/do not have the sensitive attribute “) based on the outcome of the randomiser. In contrast to Warner’s model, the answers “true/false” are replaced by innocuous answers, like a colour or a geometric figure	The model embeds the sensitive target question into *k* (= 2, 3, 4, etc.) number of innocuous unrelated questions, each with 0.5 probability). Respondents are instructed to reveal only the total number of ‘yes’ responses without revealing which ones	The model combines two questions (one sensitive target question and one innocuous question with known probability) and uses a randomiser to instruct participants on which question to answer
Protection	Combined response options	Randomised which question to answer	Randomised which question to answer	Combined responses	Randomised which question to answer
Face validity	All respondents answer the target question	A pre-determined proportion of the sample forced to say ‘yes’ or ‘no’, or instructed to answer one of the two unrelated questions	A pre-determined proportion of the sample answers the unrelated question only	All respondents answer the target question	A pre-determined proportion of the sample answers the unrelated question only
Forced ‘yes’	No	Yes	No	No	No

#### Data

Data were specified as collected between 2003 (*n* = 2) and 2022 (*n* = 1) using online surveys (*n* = 17), paper-and-pencil surveys (*n* = 11), digital (tablet computer) surveys (*n* = 3) and online and paper-and-pencil (*n* = 1), as well as online and tablet computer (*n* = 1) surveys, and an in-person interview (*n* = 1). Most data (*n* = 28) was collected at sport events.

#### Doping Operationalisation

The WADA code was applied as a definition of doping for data collection in most studies in the meta-analysis (*n* = 18) although the definitions varied across the studies with reference to “forbidden” to “prohibited” substances. In the other studies (*n* = 16), doping was operationalised as use of anabolic–androgenic steroids (AAS), selective androgen receptor modulators (SARMs), illicit drugs, prescription drugs or controlled drugs. A quarter of the included studies did not provide the exact wording of the doping-related questions used or reported prevalence in a manner inconsistent with the stated survey question. Those included exhibited considerable variation in precision, clarity and neutrality, with some displaying potential bias or judgmental framing (see Additional file 2: Table S2 for details).

Some questions employed clear and specific terminology, such as references to AAS, blood manipulation or prohibited substances by WADA, which enhances precision and comparability. However, the level of detail within these studies varies, with some studies listing specific substances (e.g. testosterone, Deca, Winstrol and Dianabol) while others use broader categories like performance-enhancing drugs or doping substances. Certain formulations of the doping question introduce implicit bias through terms like forbidden substances, illicit or illegal drugs, or violated anti-doping regulations, which suggest wrongdoing and thus may discourage honest responses due to social desirability bias, despite protections provided by indirect questioning methods.

In contrast, several studies used a more neutral phrasing, such as use of prohibited substances, thereby reducing the risk of respondents feeling accused. Some questions explicitly frame usage as intentional or knowing, which may lead to underreporting by individuals uncertain about a substance’s status, whereas the inclusion of medical exemptions (e.g. Therapeutic Use Exemption [TUE]) in some questions provides nuance. The omission of TUE considerations in other questions, however, risks conflating legitimate medical use of a prohibited substance with doping. Furthermore, some questions explicitly asking whether substances were used to enhance performance (e.g. to improve your sports performance, in order to enhance your sporting performance) could have potentially led to underreporting if athletes used substances for other or mixed reasons—such as recovery, injury management or weight loss—but not explicitly for performance enhancement.

The timeframe specified in doping-related questions also varies. While most studies focus on the past 12 months (*n* = 18), others assess lifetime/ever use (*n* = 12), past season use (*n* = 2), last 3 months use (*n* = 1), recent use (*n* = 1) or current use (*n* = 5). Five studies examine both past 12-month/season use and lifetime use, while four studies do not clearly define the timeframe used.

#### Noncompliance

The average estimated noncompliance in the included studies was 28.81% (SD 17.44, range 0.0–64.9). Less than half of the included studies (*k* = 13) estimated the magnitude of sample noncompliance, and only one study [38] estimated and corrected for motivated, self-protective noncompliance. In two studies [28, 41], the possibility of noncompliance was discussed but its magnitude or impact was not quantified. The authors of the remaining studies (*k* = 15) did not consider the potential impact of noncompliance with survey instructions. Notably, the two studies excluded for duplication of data [35, 37] also considered noncompliance for FR and SSC models respectively.

### Meta-analysis of Doping Prevalence

**Fig. 2:**
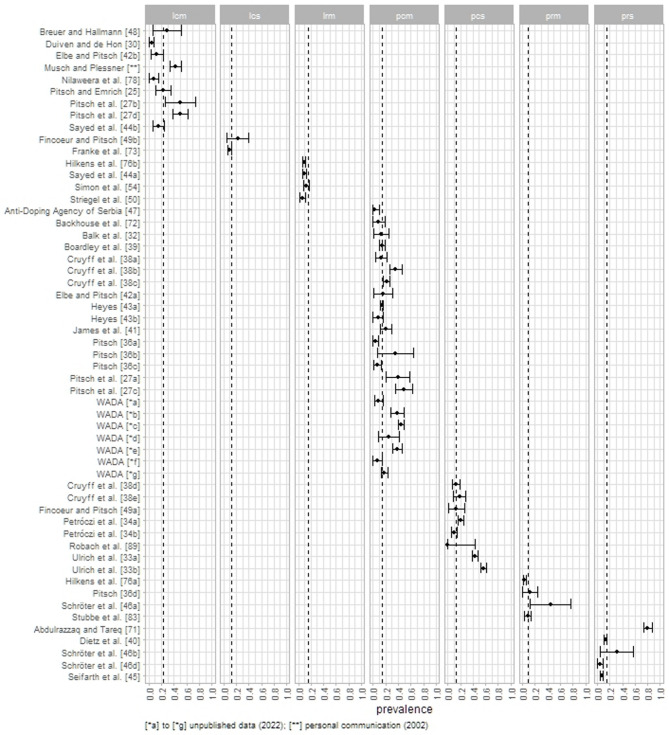
Forest plot displaying the doping prevalence (lifetime and past year) of competitive athlete and recreational sportsperson samples by sports type (multi-sport and single-sport). The filled circles indicate estimates; lines show the 95% confidence intervals; dashed lines indicate the multilevel analysis estimates. Suffixes (e.g. a, b) have been inserted to author names with multiple (e.g. lifetime and recent) estimates for nuance/separate plots to generate a single figure across the four categories.* lcm*Lifetime prevalence for competitive multi-sport,* lcs* lifetime prevalence for competitive single-sport,* lrm* lifetime prevalence for recreational multi-sport,* pcm* past year prevalence for competitive multi-sport,* pcs* past year prevalence for competitive single-sport,* prm* past year prevalence for recreational multi-sport,* prs* past year prevalence for recreational single-sport

Lifetime estimates of doping prevalence were 22.6% for multi-sport competitive athlete samples and 15.0% for multi-sport recreational sportsperson samples. Also, past year estimates of doping prevalence were 14.1% for multi-sport competitive athlete samples and 8.7% for multi-sport recreational sportsperson samples. Moreover, the lifetime prevalence estimates for single-sport competitive athlete samples and single-sport recreational sportsperson samples were 12.7% and 15.4%, respectively, whereas past year prevalence estimates were 12.7% and 15.5% for single-sport competitive athlete samples and single-sport recreational sportsperson samples, respectively. The lifetime and past year doping prevalence estimates for competitive athletes and recreational sportspersons by sports type (single-sport and multi-sport) are shown in Table 3 and Fig. 2.

**Table 3:** Doping prevalence estimates for competitive athlete and recreational sportsperson samples

Sports type	Lifetime prevalence, % (95% CI)		Past year prevalence, % (95% CI)	
	Multi-sport	Single-sport	Multi-sport	Single-sport
Competitive athletes	22.6 (14.3–33.1)	12.7 (1.9–41.7)	14.1 (8.9–21.1)	12.7 (5.6–24.5)
Recreational sportspersons	15.0 (7.1–27.2)	15.4 (1.5–55.4)	8.7 (6.0–12.2)	15.5 (5.5–33.3)

CI Confidence interval

## Discussion

We examined the prevalence of doping in competitive and recreational sport from IEM through a systematic review and meta-analysis.

### Study Characteristics

Study participants were mostly multi-sport athletes and competed at diverse levels, ranging from international levels to regional, national, local and recreational, university and school level. However, we found limited variability in the estimation models applied, with the majority of studies included in the meta-analysis applying the UQM and FR, which reflect the maturity of these models [52, 69]. Other IEM applied in the studies we reviewed, such as the SSC and the CM, have more recent history [13, 52]. Work from the WADA Prevalence Project dominantly features the Extended CM [38, 44], with limited application of the SSC [84] in earlier studies. Our finding that most data were not collected at sport events may be attributed to the bureaucratic and practical exigencies of data collection at such events [57]. Due to the focus on doping in sports and the sampling of elite athletes in many studies, it is reasonable that the WADA code was applied as a definition of doping for data collection in most studies. On the other hand, the sampling of non-competitive and recreational sportspersons in other studies may explain the application of non-WADA definitions of doping.

Overall, while many questions are well-structured, greater consistency in wording, clarity in defining key terms and neutrality in phrasing could improve the validity and reliability of survey findings. The apparent diversity in definitions and timeframes used to assess doping use reflects both the complexity of accurately measuring doping behaviour in sports and a critical issue, namely the lack of standardised definitions of doping in survey-based studies. While it is understandable that researchers tailor questions to their specific interests, leading to natural variation across studies, such inconsistencies can result in imprecise assessments, particularly when respondents must decide whether their actions qualify as doping. This lack of standardisation also complicates comparisons across studies and limits the ability to draw general conclusions about doping prevalence. For this study, we categorised any form of substance use prohibited by WADA as doping and considered current use as a subset of lifetime (ever) use.

The inclusion of intention-based language could introduce bias, as respondents may hesitate to admit that their usage was directly linked to performance enhancement, particularly given the stigma associated with doping. A more neutral and behaviourally focused approach by asking about substance use without judgements is recommended. Future research would benefit from more standardised definitions of doping and timeframes, not only in studies using indirect estimation models but in all empirical investigations of doping prevalence. Gleaves et al. [38] present specific recommendations.

### Doping Prevalence

Doping is typically detected through biological testing using urine and blood samples producing adverse analytical findings (AAF; more specifically, positive doping tests) or via longitudinal analysis of selected biomarkers (e.g. Athlete Biological Passport). Due to the clearance rate of the prohibited substances, AAFs can only indicate an incidence that is specific to a substance or group of substances, and bound by a short time window [1, 3, 58], whereas the Athlete Biological Passport is highly sensitive to potential confounding factors [59, 60] and predominantly applied in specific (endurance) sports. Prevalence estimates based on the detection of prohibited substances and/or methods are typically derived from two indicators. The first is the incidence of AAF, which consistently yields an annual rate of approximately 1% [92]. The second is the number of sanctioned anti‑doping rule violations (ADRV), representing roughly 60% of all AAF [92], plus other non-analytical ADRV such as possession, trafficking, whereabouts filing failure, refusal of sample or prohibited association. This corresponds to an estimated 0.6–0.7% of all tested athletes per year [92] and reflects a combination of intentional doping and unintentional rule violations.

In contrast, self-reports such as surveys and interviews are more suitable to assess past year/season and lifetime use of doping substances, particularly for large non-competitive samples. It is therefore not surprising that most studies included in the meta-analysis assessed past year/season and lifetime doping prevalence, although a few studies used different timeframes such as ‘current use’ or ‘past 3-month use’. Naturally, lifetime use, sometimes referred to as ‘ever use’, includes current and all past use, and their estimates are further influenced by limitations in recall accuracy. Although self‑report measures primarily reflect intentional use of prohibited substances and/or methods, the validity of prevalence estimates derived from such admissions depends on respondents’ accurate understanding of what constitutes a prohibited substance or method at the relevant time, as well as their willingness to disclose this information.

The highest estimated lifetime admitted doping prevalence rate (22.6%) for multi-sport competitive athlete samples suggests that about one of five multi-sport competitive athletes in our sample of included studies admitted to ever doping under IEM. Similarly, the highest estimated past year admitted doping prevalence rate (15.5%) for single-sport recreational sportsperson samples suggests that under IEM, about one of six of this sample in our included studies admitted to doping at least once in the past year. Here, the overlapping confidence intervals and the absence of significant lifetime and past year prevalence difference in sample type (competitive vs. recreational) and sports type (single-sport vs. multi-sport) in our multilevel model is explainable by the value of IEM in protecting respondents, thereby facilitating the generation of honest responses [13]. It is noteworthy that lifetime prevalence is naturally higher than past year/season prevalence due to the former’s wider coverage. Exploring the differences and advantages of combining both lifetime and past year questions for a single compound variable for the prevalence estimate, Sayed et al. [44] proposed the use of a multinomial model to estimate the prevalence of past year users more efficiently than the binomial model with a single question, and to create a degree of freedom necessary to test for survey instruction compliance.

Conversely, similar mistrust or confusion around the IEM procedures, as evident in the high rate (28.81%) of noncompliance in the present study, between competitive athlete and recreational sportsperson samples is another plausible explanation for the absence of prevalence differences between the two groups. Furthermore, our finding that single-sport competitive athlete samples and recreational sportsperson samples show relatively stable lifetime and past-year prevalence estimates is explainable by the value of IEM as indicated above [13]. Additional plausible explanations for this finding are the inclusion of bodybuilders [71, 76] in the single-sport recreational sportsperson sample, and the elite (international and national) sample and the anti-doping milieu of elite sports.

### Handling of Instruction Noncompliance

Among the studies included in this review, less than half addressed noncompliance with survey instructions. This is concerning because noncompliance presents the biggest threat to the validity and reliability of the IEM instrument, and therefore negatively impacts the quality of the data for prevalence estimation. The rate of assessed noncompliance in our study (28.8 ± 17.4%) is in line with those recorded in the literature where the average rate of noncompliance was estimated at 24.4% with a wide range (3.7–67.5%) [61].

The interpretation and handling of noncompliance in the studies included in this review presented a diverse picture. Some authors interpreted this noncompliance as cheating [46, 49, 83]. Alternatively, in other studies [36, 37, 48, 79], the authors reported the proportion of honest ‘no’ responses, thus leaving the combination of honest yes (admitted doping) and survey noncompliers open to interpretation. The authors of other studies [26, 42, 74] assumed that survey noncompliance is motivated by self-protective cheating, and thus reported the maximum value of noncompliance as the possible upper limit of the discriminating behaviour, which resonates with a similar interpretation by Ostapczuk et al. [62]. In prevalence estimations using the SSC model [34, 47, 72], the authors reported the estimated noncompliance to be proportionate to admitted dopers and honest non-dopers.

Several plausible hypotheses can be devised about how dopers and non-dopers might respond to a survey and whether motivated as well as nonmotivated noncompliance is equally present among dopers and non-dopers—for example Ulrich et al. [35]—but these assumptions, to date, lack empirical evidence. Cruyff et al. [38] addressed self-protective noncompliance based on empirical evidence from a series of studies and literature [63], but noted the lack of a test for inattentive noncompliance in the CM. Furthermore, Nepusz et al. [64] proved that the independent model, where noncompliance is assumed to be independent of being guilty, cannot be statistically outperformed by a dependent model that assumes that noncompliance and the guilty attributes are not independent. For example, the subsample of guilty has a higher degree of noncompliance because it combines nonmotivated careless responding with motivated self-protective lying while the non-guilty group is only affected by the former. Unfortunately, the SSC model cannot help with the decision about which assumption describes actual noncompliance better because for every dependent model there is an equally fitting independent model.

The magnitude of noncompliance in the present study, as well as in the broader IEM literature [61], highlights that the weakness of indirect estimation models and self-reports in general is the unknown probability of dishonest and inattentive (random) responding—thus the human element. Naturally, attention turned to understanding, comprehension and trust (e.g. [65–67]), and self-protective cheating (e.g. [68, 69]). However, as much as providing a safe survey environment addresses respondents’ fear of exposure, it does not necessarily motivate full engagement with the survey. Previous studies also showed that random responding is present in applications of IEM (e.g. [70, 85]) and offered various analytical solutions to correct estimations for survey instruction noncompliance [e.g. 85–89]. At maximum prevalence of inattentive, random responding (i.e. all respondents answer randomly), the estimated prevalence rate approaches 50%, whereas its impact is negligible if the proportion of random responding is low [70].

### Emerging Challenges

Indirect estimation models require complex transformations of raw data into prevalence estimates, a process that is far from straightforward. These models rely on multiple assumptions regarding the distribution of innocuous or unrelated responses, which serve the purpose of protecting both respondents and researchers, and play a crucial role in estimating the affirmative answers to the sensitive prevalence question. Additionally, prevalence estimates depend on assumptions about respondent behaviour, as research has consistently shown that IEM are prone to noncompliance with survey instructions [61, 87, 88], which can markedly affect the validity of the estimates.

These methodological challenges extend to secondary data analysis, an increasingly accepted and encouraged research practice, posing two critical issues for meta-analyses of indirect estimates. The first challenge concerns the handling of multiple outputs derived from the same dataset, such as duplicated estimates published in different languages or by different authors [24–28, 28, 29]. The second challenge arises from the substantial influence of modelling assumptions on prevalence estimates. Studies have demonstrated that varying these assumptions can lead to markedly different estimates [33–35, 38, 90], often without robust empirical evidence for preferring one set of assumptions over another. It remains unclear how to systematically address multiple outputs that yield different prevalence estimates for the same population, whether selecting one is a right approach, and if so, what objective criteria can be set for selecting among conflicting estimates and strategies for mitigating the impact of assumption-driven variability on overall findings.

### Strengths, Limitations, and Implications

To our knowledge, the present study is the first systematic review on the prevalence of doping in sport using IEM. The multi-lingual (English, German, Dutch, French, Russian, and Spanish) literature search and multi-faceted meta-analysis are other strengths of our study. Study limitations include the predominance of European and German samples, the use of the average estimate for the CDM and sample heterogeneity (recreational, competitive, bodybuilders etc.), all of which limit generalisability to a specific population of sportspersons. Here, although competitive athletes can be stratified as elite (e.g. international; Balk et al. [32]) and non-elite (e.g. schoolboy; Backhouse et al. [72]), this stratification was not feasible in our study due to data sparsity. Future research is encouraged to incorporate this distinction to enable more nuanced estimates of doping prevalence among competitive athletes.

Also, due to the mode of addressing survey noncompliance in some studies using the CDM, by either combining the estimated admitted prevalence and noncompliance as ‘potential use’ of doping or reporting honest ‘no sayers’, we used the midpoint of the lower and upper bound as the point estimate of doping use of the combined honest users and noncompliers in the meta-analysis. Another limitation is the susceptibility of past year and lifetime prevalence estimates to recall bias due to their retrospective nature. It is plausible that the latter is more applicable to recreational sportspersons than to competitive athletes due to cognisance of the severe consequences of using prohibited substances in competitive sport.

With the preponderance of included studies conducted in European countries, particularly Germany, more research is recommended in other regions and countries, particularly among non-Western, Educated, Industrialised, Rich, and Democratic (WEIRD) [91] samples. Also, given the limited variability in the estimation models with the majority of studies applying the UQM and FR or SSC, more empirical applications of other IEM, such as the Extended CM, and Kuk’s Design, are encouraged. Here, recent critique of the SSC [35] is noteworthy in model selection. Future research on doping prevalence in sports should carefully evaluate the most effective data collection methods, taking into consideration the trade-offs between event-based data collection and personal invitations via email. Additionally, the choice between single versus multisport events and national versus international settings should be considered. Key factors to consider include access to events and participants, data protection concerns, participant convenience and motivation and the potential impact on the reputation of specific sports or countries, particularly in studies focused on single-sports or nations when results are made public. As noted previously, a more detailed exploration of IEM validity and reliability as well as recommendations for future studies applying IEM to doping prevalence (e.g. IEM selection, data collection, analysis and dissemination) are warranted. We are addressing this in a separate article, along with recommendations for conducting and reporting IEM-based doping prevalence studies.

## Conclusions

Our systematic review and meta-analysis provides unique in-depth perspective into the application of IEM to doping prevalence. The included empirical studies have been preponderantly conducted in European countries, particularly Germany. Studies mostly assessed admitted doping for the past year/season, and were based mainly on the UQM and FR models with samples consisting of largely multi-sport competitors of various levels. Pooling IEM estimates, about one of five multi-sport competitive athletes in our sample of included studies admitted to ever doping whereas about one of six single-sport recreational sportspersons admitted to doping in the past year. Additionally, lifetime prevalence is relatively higher among multi-sport competitive athlete samples whereas past-year prevalence is relatively higher among single-sport recreational sportsperson samples. Furthermore, single-sport (competitive or recreational) is associated with relatively stable lifetime and past-year prevalence estimates.

## Supplementary Information

Additional file 1: Table S1. Characteristics of studies estimating doping prevalence in sport using indirect estimation models.

Additional file 2: Table S2. Doping definition table.

## Data Availability

The manuscript has data included as electronic supplementary material.

## Abbreviations

AAF: Adverse analytical findings
AAS: Anabolic–androgenic steroids
ADRV: Anti‑Doping Rule Violations
CDM: Cheater Detection model
CM: Crosswise Model
FR: Forced Response
IEM: Indirect estimation models
PRISMA: Preferred Reporting Items for Systematic Reviews and Meta-Analyses
PWG: Prevalence Working Group
RRT: Randomised response technique
SARMs: Selective androgen receptor modulators
SSC: Single sample count
TUE: Therapeutic Use Exemption
UQM: Unrelated Question Model
WADA: World Anti-Doping Agency
WEIRD: Western, Educated, Industrialised, Rich, and Democratic

## Footnotes

1 World Anti-Doping Agency. Doping Prevalence Working Group. Petróczi A, De Hon O, Saugy M, Cruyff M, Sagoe D, Gleaves J. Interim report (unpublished report). Montreal; 2022.

2 Musch J, Plessner H. A randomized response investigation of the prevalence of doping. 2002. Unpublished manuscript used with authors permission.
